# CXCL2-mediated ATR/CHK1 signaling pathway and platinum resistance in epithelial ovarian cancer

**DOI:** 10.1186/s13048-021-00864-3

**Published:** 2021-09-03

**Authors:** Sipei Nie, Yicong Wan, Hui Wang, Jinhui Liu, Jing Yang, Rui Sun, Huangyang Meng, Xiaolin Ma, Yi Jiang, Wenjun Cheng

**Affiliations:** Department of Gynecology, the First Affiliated Hospital of Nanjing Medical University, Nanjing, 210029 Jiangsu China

**Keywords:** Epithelial ovarian cancer (EOC), Platinum-resistance, Chemokine, CXCL2

## Abstract

Tumor microenvironment and chemokines play a significant role in cancer chemoresistance. This study was designed to reveal the important role of CXCL2 in platinum resistance in epithelial ovarian cancer (EOC). Differently expressed (DE) genes were screen out based on analysis of GSE114206 dataset in GEO database. The expression of DE chemokines was further validated in platinum- resistant and sensitive EOC. Cell viability assay and cell apoptosis assay were performed to explore the roles of CXCL2 in EOC. Cell stemness characteristics and the signaling pathway regulated by CXCL2 were also investigated in this study. As the results showed, CXCL2 was identified up-regulated in platinum-resistant EOC. The functional assays showed overexpressing CXCL2 or co-culturing with recombinant human CXCL2 promoted cell resistance to cisplatin. Conversely, knocking down CXCL2 or co-culturing with neutralizing antibody to CXCL2 increased cell response to cisplatin. CXCL2 overexpressing maintained cell stemness and activated ATR/CHK1 signaling pathway in EOC. Moreover, we further demonstrated that CXCL2-mediated resistance to cisplatin could be saved by SB225002, the inhibitor of CXCL2 receptor, as well as be rescued by SAR-020106, the inhibitor of ATR/CHK1 signaling pathway. This study identified a CXCL2-mediated mechanism in EOC platinum resistance. Our findings provided a novel target for chemoresistance prevention in EOC.

**Supplementary Information:**

The online version contains supplementary material available at 10.1186/s13048-021-00864-3.

## Backgrounds

Epithelial ovarian cancer (EOC) is the first cause of gynecological malignancy-related death [1]. At present, platinum-based chemotherapies were recommended as the first-line chemotherapeutic regimens for EOC. Although the tremendous progress has made in comprehensive therapies, the survival rate of EOC patients with advanced tumor remains poor even in high‐resource countries such as the United States and Canada [2]. Though initial response rates of platinum-based regimens are 60–80%, majority of EOC patients acquire platinum resistance during treatment. Platinum resistance is one of the primary causes of subsequent relapses and metastasis [3]. Thus, platinum resistance remains an urgent challenge for EOC patients, but the mechanism remains unidentified. There is a lack of effective approach to against platinum resistance in malignancies. Therefore, revealing the molecular mechanism contributing to platinum resistance in EOC and exploring therapeutic targets are clinical significances.

Chemokines contain a group of about 50 small (8– 14 kDa) secreted proteins. These molecules regulate cell biological processes, including in malignancies initiation and progression [4, 5]. These secreted proteins work by interacting with the corresponding receptors, a family of about 20 seven-transmembrane G-protein-coupled receptors [6]. Previous studies have suggested chemokines and their corresponding receptors were involved in malignancies progression mainly in the three mechanisms: attracting cancer cells for metastasis; mobilization of hematopoietic cell populations from the bone marrow to colonize at the tumor site and regulate tumor processes; acting as growth factors and supporting tumor growth through an autocrine pathway [6, 7]. Increasing evidences have supported that using immune checkpoint inhibitors that targeting chemokines and their receptors becomes a novel approach of cancer therapy [8–10]. The anti-CCR4 monoclonal antibody and the CXCR4 receptor inhibitor are already in the clinical practice for hematological malignancies [11–15]. CXCL10 was suggested as an immune checkpoint molecule in cancer, displaying a positive autocrine effect and directly suppressing tumor growth[16]. CXCL17 expression was that associated with lung cancer and hepatic cancer[17]. In addition, recently studies also proved chemokines and their receptors participate in the cancer chemoresistance. Ren et al. demonstrated CXCR3-mediated AMPK signaling pathway contributed to metabolic alteration during the chemoresistance to agent-sorafenib in hepatocellular carcinoma [18]. Zhang et al. identified CXCL13 was involved in 5-Fu resistance in colorectal cancer [19]. These studies suggested that chemokines and their receptors might be potential novel therapeutic targets of cancer chemoresistance.

Thus, the present study devoted to exploring the role of chemokines in platinum-resistant EOC and investigate the biological function and underlying regulation mechanism of the candidate chemokine. Currently, gene expression profiles have been increasingly used to identify candidate significant genes in various diseases, especially in malignancies [20]. Public genomics data repositories provided powerful systems biology approaches to detect the association between genes and cancer. In the present study, we devoted to exploring the role of chemokines in platinum-resistant EOC. We used bioinformatics methods to screen our differentially expressed (DE) chemokines by comparing gene expression profiles based on platinum sensitivity status and ADP-ribose polymerase (PARP) levels. Candidate DE chemokines were next validated in platinum-resistant and sensitive EOC samples and cells.

## Materials and Methods

### Differentially expressed genes (DEGs) screening in EOC with platinum resistance

The gene expression profile was obtained from GSE114206 dataset of GEO database. GSE114206 dataset contained 6 low PARP and platinum-resistant EOC samples and 6 high PARP and platinum-sensitive EOC samples. We use the “limma” R package to screen the DEGs between the platinum- resistant and sensitive EOC samples. Adjusted *P*-value < 0.05 and |log2fold change (FC)|> 1, were chosen as the cut-off threshold. Candidate DE chemokines were visualized by “circos” package (https://www.omicstudio.cn/tool).

### Patients and clinical sample collection

All patients signed informed consent before using clinical specimens, and the use of specimens for this study has been proved by the ethics committee of the First Affiliated Hospital with Nanjing Medical University. All the ovarian cancer tissues and serum samples were obtained from patients diagnosed and underwent surgery in the First Affiliated Hospital with Nanjing Medical University between January 2015 to January 2020. Surgical specimens were immediately at − 80 °C for nucleic acid and protein extraction. Patient who did not achieve the clinical complete response in the initial therapy or whom with recurrent tumor within 6 months was identified as an primary resistance case [21]. Due to the secondary operation is not recommended in patients with recurrent resistant tumor, collected samples in our study were limited (15 platinum-sensitive and 9 platinum-resistant EOC samples). Meanwhile, serum samples of 6 platinum-resistant and 9 platinum-sensitive EOC patients were also collected.

### Cell culture and chemicals

HO8910 and A2780 are platinum-sensitive EOC cells. By exposure to increasing concentration of cisplatin, the cisplatin-resistant cells A2780-DDP, HO8910-DDP were constructed in our study for further research. FBS (CLRAK Bioscience) and 1% penicillin/streptomycin (Sigma-Aldrich) at 37℃ supplied with 5% CO2.

### Plasmid and siRNA transfection

The plasmid encoding the transcript of CXCL2 (oeCXCL2) and the negative control (oeNC) was synthesized by Tsingke (Nanjing, China). The siRNA targeted CXCL2 (siCXCL2) and the negative control (siNC) were purchased from GemmaPharma (Shanghai, China). Cells were transfected by Lipofectamine 3000 Transfection Reagent (L3000150, Invitrogen) for 24–48 h. The CXCL2 siRNA sequence was described in the previous study [22].

### Cell viability assay

About 6000–8000 preprocessed EOC cells were plated in 96-well plates, and incubated in a series of cisplatin concentration (0, 5, 10, 15 and 20 μM) (Sigma-Aldrich) for 48 h. Cell viability was qualified by Cell Counting Kit-8 (CCK8) (Vazyme) under the manufacturer's instructions. A microplate reader (TECAN, Infinite M200 PRO) was applied to measure the absorbance at 450 nm, and the cell’ IC_50_ were analyzed in Graphpad 8.0.

### RNA extraction and quantitative Real-time PCR (qRT-PCR)

According to the manufacturer's instructions, the total RNA of cultured cells and EOC tissues was extracted with Trizol (Invitrogen). cDNA HiScript Q RT SuperMix for qRT-PCR (Vazyme) were used to prepare cDNA. qRT-PCR was performed under the instructions of SYBR Green PCR Kit (Vazyme). The sequences of gene primers used for qRT-PCR were synthesized by Tsingke (Nanjing, China) (Supplementary Table 1). The mRNA expression level was normalized to GAPDH to calculate the relative gene expression.

### Western blot assay

Firstly, cultured cells were lysed in RIPA buffer (Beyotime) with protease inhibitor (Beyotime). Proteins were then extracted and quantified. Western blot assay was performed by the protocol that we previously reported [23]. The reagents and antibodies were shown in Supplementary Table 2.

### Enzyme-linked immunosorbent assay (ELISA)

Serum samples of platinum-resistant and platinum-sensitive patients and cell culture supernatants were respectively extracted for cytokine analysis. Human CXCL2 ELISA kits were purchased from Hengyuan Biological Co. Ltd (Shanghai, China), and ELISA assay was performed as the manufacturers’ instructions. Each sample was duplicated.

### Cell apoptosis assay

EOC cells were plated in 6-well plates and treated with cisplatin (5 μM/10 μM) for 48 h. Then, 2 × 10^4^ cells and the cultural supernatant were collected for apoptosis assay. FITC Annexin V (5 μl) and propidium iodide (5 μl) (BD Biopharmingen) were used to stain the collected cells by suspending in 300 μl of binding buffer for 15 min in the dark. Cell apoptosis was eventually detected by a flow cytometer (FACScan; BD Biosciences) with Cell Quest software (BD Biosciences).

### Statistical analysis

Each sample was analyzed based on repeated results at least three times and analyzed in Graphpad 8.0. A standard Student's t-test determined the statistical significance of differences between the two groups. Differences at *p* < 0.05 were regarded as statistically significant (*), ones at *p* < 0.01(**), *p* < 0.001(***) or *p* < 0.0001(****) was considered higher statistical significances.

## Results

### CXCL2 is up-regulated in platinum-resistant EOC

**Fig. 1:**
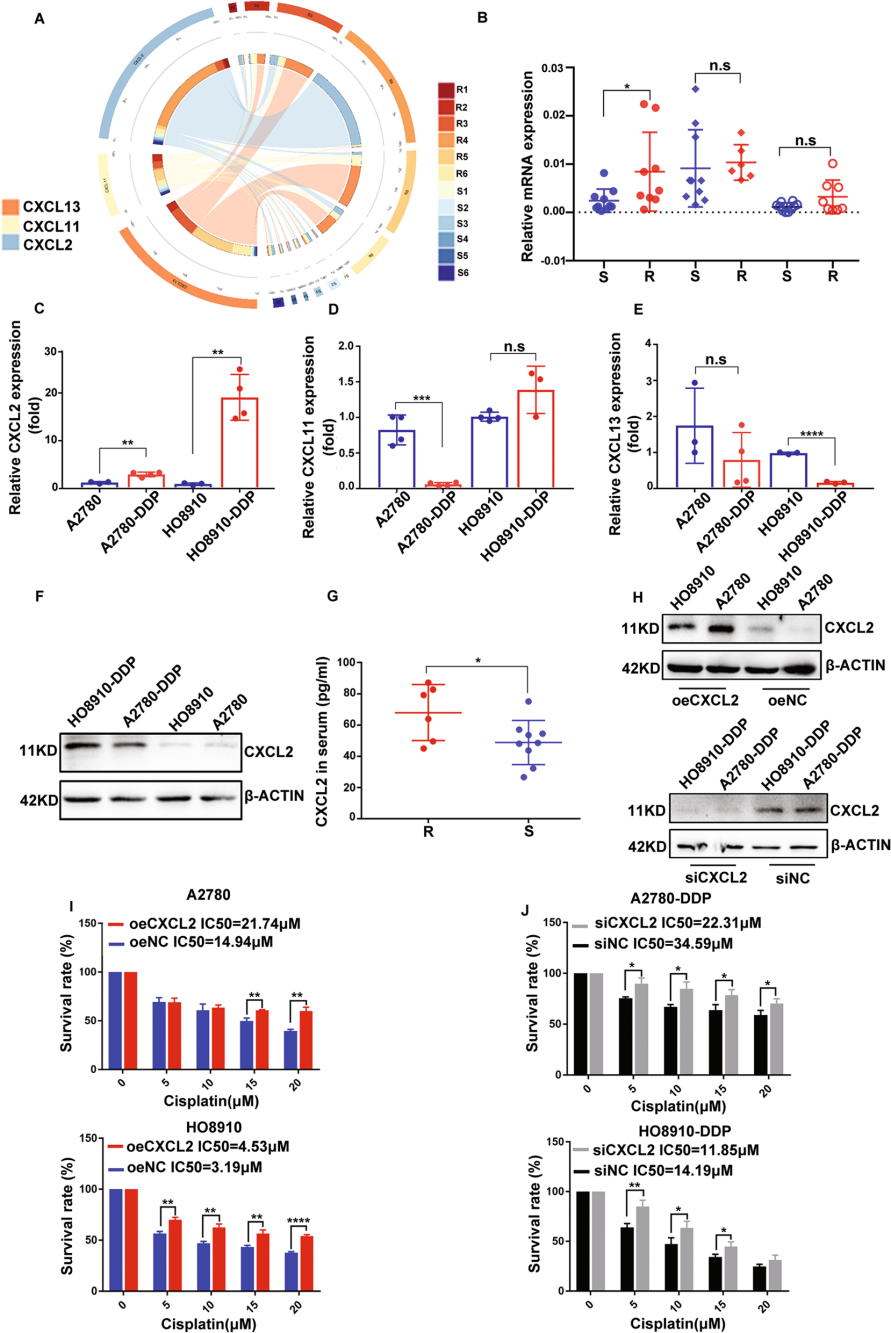
CXCL2 overexpression promotes cell resistance to cisplatin in EOC. **A**. CXCL2, CXCL11 and CXCL13 were identified as DEGs in GSE114206. **B** The mRNA expression in platinum-resistant (n = 9) compared to platinum-sensitive patients (n = 15). **C-E** The mRNA expression in cisplatin-sensitive and resistant EOC cell lines. **F** The CXCL2 protein level in cisplatin-sensitive and resistant EOC cell lines. **G** CXCL2 levels in serum detected by ELISA assay. **H** Overexpressing and knocking down efficiencies of transfection. **I** The cell viability assay showed overexpressing CXCL2 significantly increased IC_50_ in EOC cells. **J** The cell viability assay showed knocking down CXCL2 significantly decreased IC_50_ in cisplatin-resistant EOC cells. Abbreviation: DEG: differentially expressed genes, R: resistant samples; S: sensitive samples

By analyzing the gene expression profiles of GSE114206 dataset, we identified 1269 up-regulated DEGs and 1140 down-regulated DEGs (*P* < 0.05, |log2FC > 1). Among these DEGs, DE chemokines including CXCL2, CXCL11 and CXCL13 were screened out (Fig. 1A). By further validating mRNA expression level of CXCL2, CXCL11 and CXCL13, we found CXCL2 was up-regulated in platinum-resistant EOC samples (Fig. 1B) and cisplatin-resistant EOC cells (Fig. 1C-F). Additionally, CXCL2 was also found up-regulated in serum of platinum-resistant EOC patients (Fig. 1G).

### CXCL2 overexpression promotes cell resistance to cisplatin

After transfecting with plasmids, A2780 and HO8910 with CXCL2 overexpressing and the negative control were constructed (Fig. 1H). Then, cell viability assays showed that CXCL2 overexpression remarkably promoted cancer cell chemoresistance in cisplatin-sensitive EOC cells (Fig. 1I). Oppositely, the cell viability assay illustrated a decreased IC_50_ in cisplatin-resistant EOC cells with CXCL2 knocking down (Fig. 1J).

### CXCL2 promotes cell resistance to cisplatin in EOC by an autocrine mechanism

**Fig. 2:**
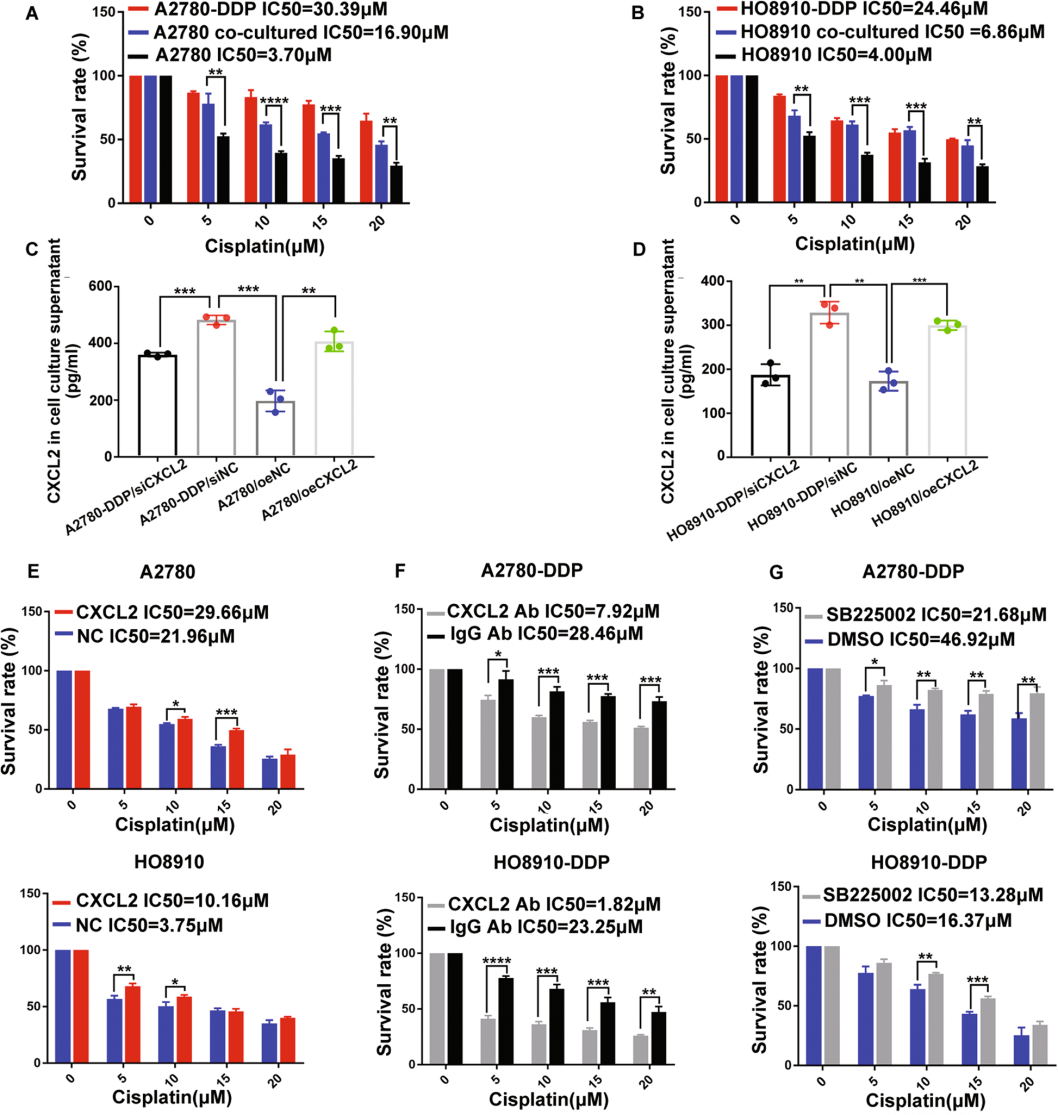
CXCL2 promotes cell resistance to cisplatin in EOC by an autocrine mechanism. **A** and **B**) IC_50_ increased in co-cultured EOC cells. **C** and **D** CXCL2 expression level in cell culture supernatant. **E** IC_50_ in EOC cells respectively incubating with recombinant CXCL2 (CXCL2, 50 ng/ml) PBS (NC) for 6 h. **F** IC_50_ in EOC cells respectively incubating with CXCL2 neutralizing antibody (CXCL2 Ab) (5 µg/mL) and the control IgG antibody (IgG Ab) for 6 h **G** IC_50_ in EOC cells respecectively pretreating with CXCR2-specific inhibitor, SB225002 (10 μM) and DMSO for 6 h

We firstly used culture supernatant of A2780-DDP and HO8910-DDP cells to incubate A2780 and HO8910 respectively. The result showed co-cultured cells behaved higher resistance to cisplatin (Fig. 2A and B). Meanwhile, we found CXCL2 was up-regulated in culture supernatant of cisplatin-resistant EOC cells (Fig. 2C). CXCL2 in culture supernatant was also consistently regulated by overexpressing or knocking down CXCL2, which suggested CXCL2 in the tumor microenvironment (TME) might be regulated by autocrine mechanism (Fig. 2D). Moreover, the addition of recombinant CXCL2 promoted cell resistance to cisplatin in A2780 and HO8910 (Fig. 2E).Oppositely, incubating with CXCL2 neutralizing antibody weakened the chemoresistance in A2780-DDP and HO8910-DDP (Fig. 2F). These findings showed that CXCL2 up-regulation in the cell culture environment promoted cell resistance to cisplatin. In addition, we further investigate whether blocking CXCR2, the receptor of CXCL2 could inhibiting cisplatin resistance. By using SB225220, the inhibitor of CXCR2, to incubate A2780-DDP and HO8910-DDP, we found the IC_50_ in cisplatin-resistant EOC cells was also decreased (Fig. 2G).

### CXCL2 inhibits cell apoptosis and maintains cell stemness in EOC

**Fig. 3:**
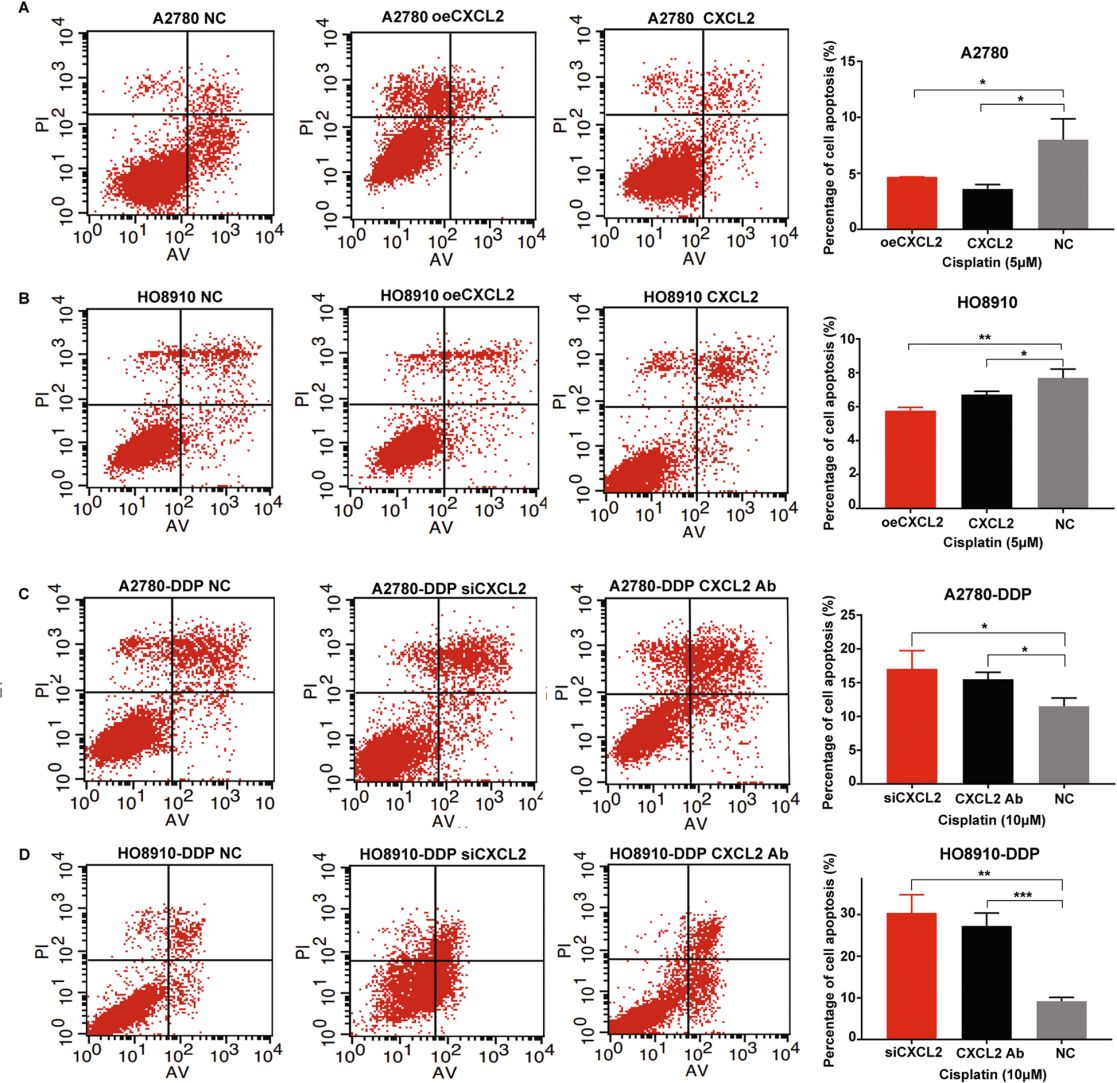
CXCL2 overexpression inhibites cell apoptosis induced by cisplatin. **A** and **B** The cell apoptosis rate increased in cisplatin-senstive cells with CXCL2 overexpressing or incubating with recombinant CXCL2 (CXCL2, 50 ng/ml) for 48 h. **C** and **D** The cell apoptosis rate decreased in cisplatin-resistant cells with CXCL2 knocking down or incubating with CXCL2 neutralizing antibody (5 µg/mL) for 48 h

**Fig. 4:**
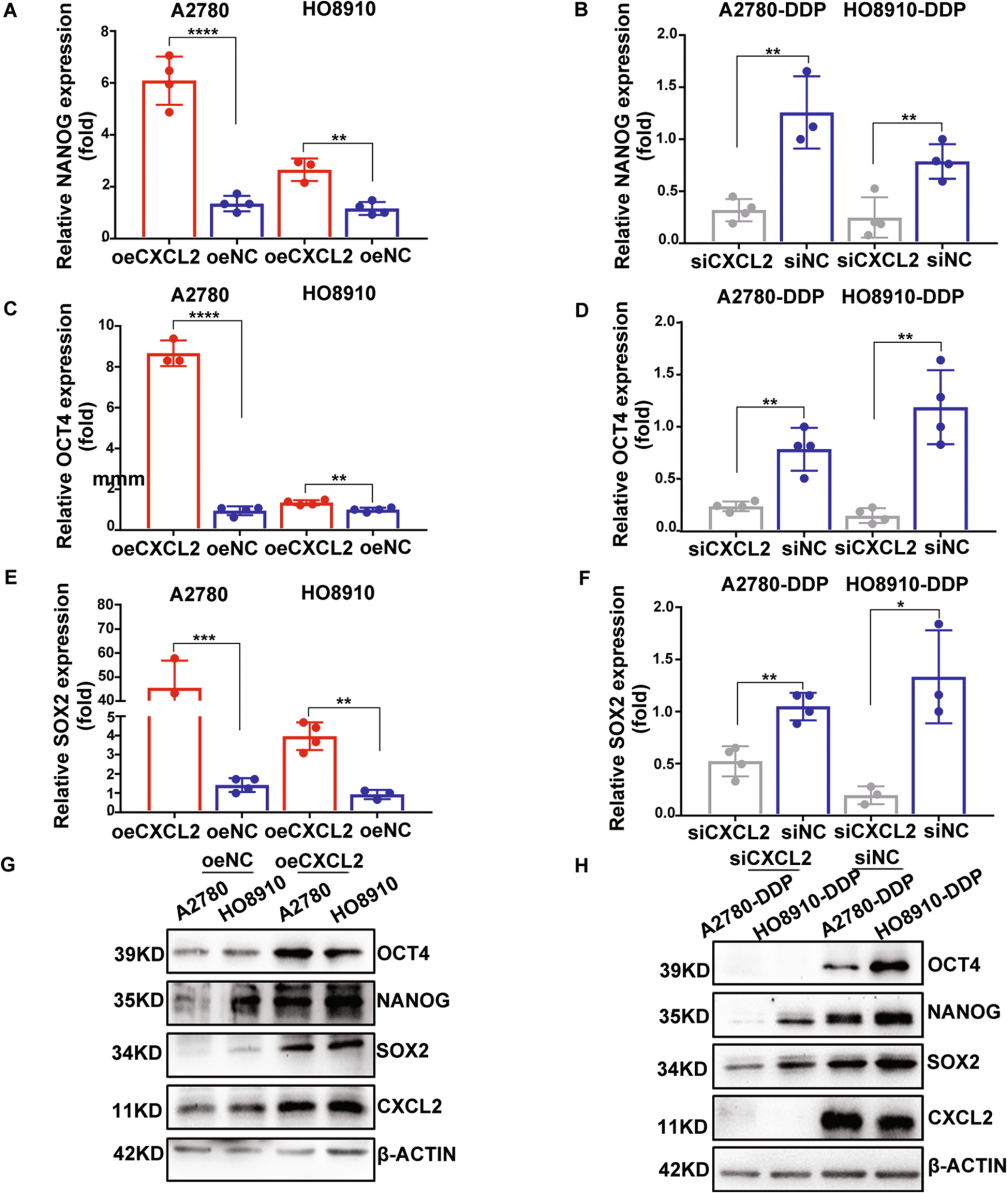
CXCL2 overexpression maintains cell stemness in EOC. **A** and **B**. NANOG mRNA level was positively regulated by CXCL2. **C** and **D** OCT4 mRNA level was positively regulated by CXCL2. **E** and **F** SOX2 mRNA level was positively regulated by CXCL2. **G** and **H** The results of Western-blot assay showed CXCL2 maintained cell stem characteristics

The results of cell apoptosis assay showed that the cell apoptosis rate was decreased in cells with CXCL2 overexpressing or cells incubating with recombinant CXCL2 (Fig. 3A and B). On the contrary, cells with CXCL2 knocking down or cells incubating with CXCL2 neutralizing antibody facilitated cell apoptosis after treating with cisplatin (Fig. 3C and D). Meanwhile, we found the cell stemness characteristic, including Nanog, SOX2 and OCT4, was also increased in cells with CXCL2 up-regulation, while decreased in cells with CXCL2 down-regulation (Fig. 4).

### CXCL2 promotes cell resistance to cisplatin in EOC by mediating ATR/CHK1 signaling pathway

**Fig. 5:**
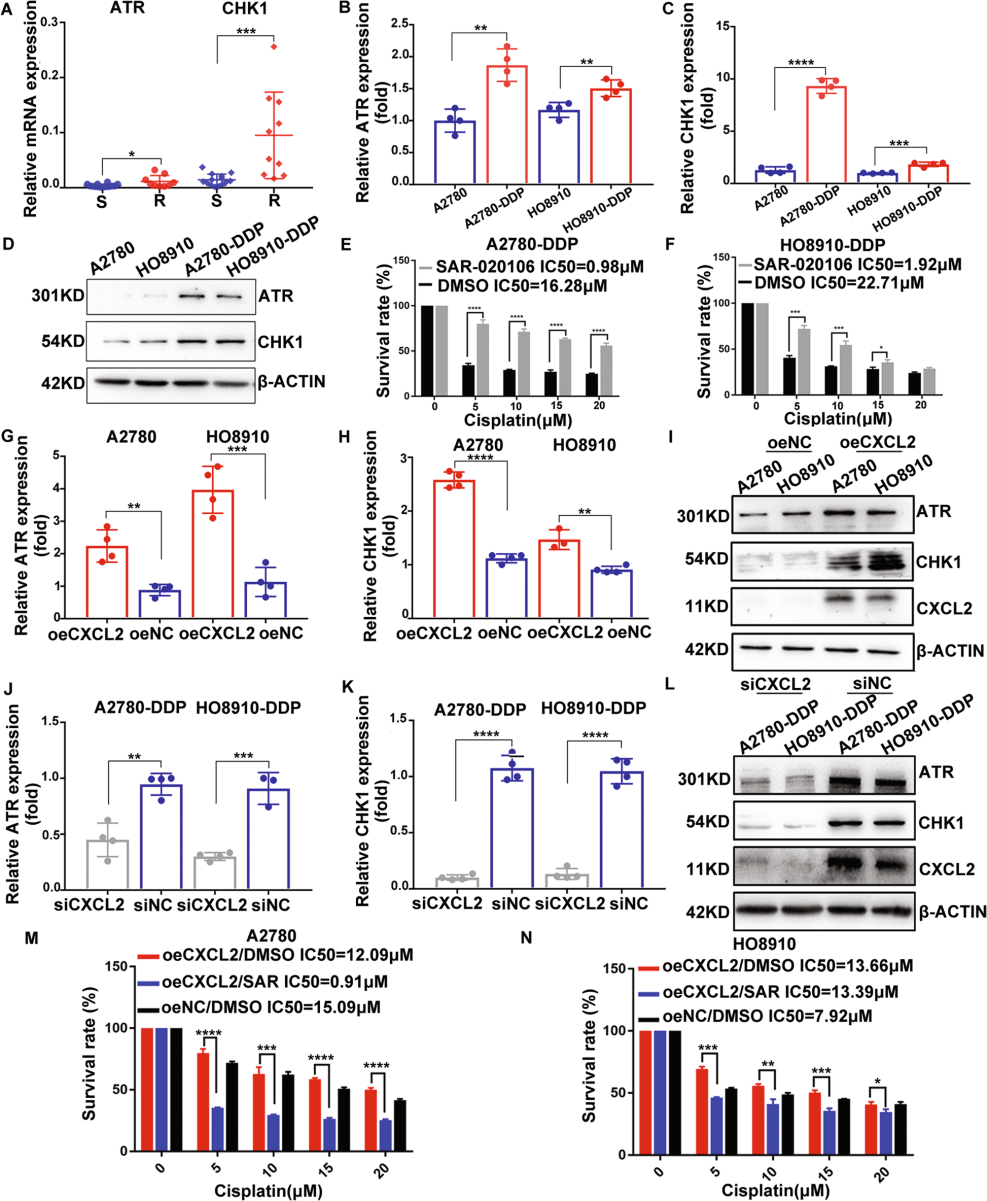
CXCL2 promotes cell resistance to cisplatin in EOC by mediating ATR/CHK1 signaling pathway. **A**-**D** ATR and CHK1 were found up-regulated in platinum-resistant EOC tissues and cell lines. **E** and **F** The addition of CHK1 inhibitor, SAR-020106 (1 μM, pretreated for 6 h) weakened chemoresistance in cisplatin-resistant cells. **G-I** ATR and CHK1 expression were found up-regulated in cells with CXCL2 overexpression. **J-L** ATR and CHK1 expression were found down-regulated in cells with CXCL2 knocking-down. **M** and **N** The addition of SAR-020106 alleviated the chemoresistance in EOC cells with CXCL2 overexpressing. Abbreviation: R: resistant samples; S: sensitive samples; SAR: SAR-020106

Given that the ATR/CHK1 signaling pathway has been proved to play a significant role in DNA damage repairment (DDR) and resulte in cancer chemoresistance. We speculated whether CXCL2 meidated cell resistance to cispaltiin in the ATR/CHK1 signaling pathway. The results showed that ATR and the downstream molecule CHK1 were up-regulated in platinum-resistant EOC samples and cells (Fig. 5A-D). To investigate the effect of ATR/CHK1 signaling pathway activation on EOC platinum resistance, we used SAR-020106, the inhibitor of CHK1, to incubate platinum-resistant cells. The results showed the addition of SAR-020106 weakened the chemoresistance in cisplatin-resistant EOC cells (Fig. 5E and F). Our findings further showed that ATR and CHK1 expressions were consistently up-regulated in cells with CXCL2 overexpressing, while down-regulated in cells with CXCL2 knocking down (Fig. 5G-L). We also analyzed the correlation of CXCL2 and ATR/CHK1 gene expression based on GSE114206 dataset and The Cancer Genome Atlas (TCGA) database. However, the results of correlation analyses showed no statistical significance (Supplementary Fig. 1). Moreover, rescue assays were performed to further demonstrate the effect of CXCL2 mediating-activation of ATR/CHK1 signaling pathway in EOC platinum resistance. The rescue assays displayed the addition of SAR-020106 alleviated the chemoresistance in EOC cells with CXCL2 overexpression (Fig. 5M and N).

## Discussions

TME significantly influences therapeutic response and clinical outcomes of malignancies. According to previous studies, multiple chemokines in TME were demonstrated to be associated with tumor growth, metastasis and cancer cell stemness, which contribute to cancer chemoresistance [6, 24, 25]. In the present study, we devoted to exploring the chemokines involved in EOC resistance to platinum. As a result, we identified candidate chemokines by bioinformatics analysis and identified that the CXCL2 was notably up-regulated in platinum-resistant EOC. CXCL2 overexpression was then demonstrated to promote cancer cell chemoresistance in EOC. The CXCL2 overexpression was further found to inhibit cell apoptosis rate induced by cisplatin and maintain cell stemness in EOC. Mechanistically, we found that CXCL2 promotes EOC cell resistance to cisplatin by mediating ATR/CHK1 signaling pathway.

As the results illustrated, we demonstrated that CXCL2 was up-regulated in platinum-resistant EOC, and itpromotes cell resistance to cisplatin. CXCL2, known as growth-related oncogene‐2/‐β or macrophage inflammatory protein‐2α, is 90% identical in amino acid sequence [26]. CXCL2 works via the corresponding receptor, CXCR2, and the CXCL2/CXCR2 axis have been extensively studied in malignancies. Previous studies reported CXCL2/CXCR2 axis played a significant role in promoting neutrophils and endothelial cells' chemotaxis, resulting in tumor growth, angiogenesis, chemoresistance, and transformation [27–29]. Makoto et al. suggested omental adipocytes triggered gastric cancer cells proliferation, migration and angiogenesis induction by CXCL2 secretion [30]. Monocytes-derived CXCL2 and CXCL8 are the main causes in regulating neutrophils' recruitment into TME of hepatocellular carcinoma, which could inhibit cancer cell apoptosis [31]. The previous study has also suggested that CXCL2 concentration was up-regulated in the serum of ovarian adenocarcinoma patients with chemoresistance. CXCR2 overexpression has been demonstrated to promote ovarian cancer progression [32, 33]. However, there is a lack of research about the role of CXCL2 and the regulation mechanism in platinum-resistant EOC. Herein, this study systematically explored the molecule biological function and the underlying mechanism of CXCL2 in platinum-resistant EOC. CXCL2 was proved to protect cell apoptosis from cisplatin treatment and maintain cancer cell stemness, which might result in cell chemoresistance phenotype.

Currently, the formation of DDP-DNA complex, which inhibited DDR is considered as one of the most classical mechanism of cisplatin anti-tumor action [34]. One of the practical approaches to intervene in the DDR process of platinum-resistant cancer is interfering with cell cycle checkpoint signaling pathway [35]. In the present study, CXCL2-mediated ATR/CHK1 signaling pathway was found to promote platinum resistance in EOC. ATR and its downstream kinase CHK1 can be activated by DNA damage and DNA replication stress [36, 37]. Activating ATR/CHK1 signaling pathway results in cell cycle arresting, allowing time for DNA replication and corrected DNA repairment [38]. Besides, activating ATR/CHK1 signaling pathway stabilizes replication forks and prevents collapse into DNA double-strand breaks [39, 40]. Those functions of ATR/CHK1 signaling pathway provide potential therapeutic targets to overcome cancer therapeutic resistance. ATR/CHK1 inhibitors have been designed and applied singly or paired with radiotherapy and chemotherapy in preclinical and clinical studies, including in EOC [41]. Huntoon et al. suggested that inhibiting ATR/CHK1 broadly sensitizes cancer cells to chemotherapy, which is independent of BRCA status in ovarian cancer [42]. Our findings suggested a novel regulation mechanism of CXCL2 in EOC platinum resistance by mediating ATR/CHK1 signaling pathway. However, by analyzing the gene data of GSE114206 dataset and TCGA database, it showed no statistically significant in correlation of CXCL2 and ATR/CHK1 expression in ovarian tumor. These results might be caused by the complex gene regulation network in cancer, and the interactions between genes may not be singular.

## Conclusions

In summary, this study revealed the crucial role of CXCL2 and demonstrated a new regulatory mechanism of CXCL2-mediated ATR/CHK1 signaling pathway in platinum-resistant EOC. Our findings suggest that blocking the role of CXCL2 and its regulatory signaling pathways represents a potential approach to overcome platinum resistance in EOC.

## Supplementary Information

**Additional file 1: Supplementary Figure 1**. The correlation of CXCL2 and ATR/CHK1 gene expression in EOC. (A) The correlation analyses of CXCL2 and ATR/CHK1 based on gene expression profile of GSE114206 showed no statistical significance. (B and C)The correlation analyses of CXCL2 and ATR/CHK1 based on TCGA database also showed no statistical significance. Abbreviation: TCGA: The Cancer Genome Atlas.

**Additional file 2: Supplementary Table 1: **The sequences of gene primers used for qRT-PCR.

**Additional file 3: Supplementary Table 2.**

## Data Availability

They could be achieved upon reasonable request to the authors.

## Abbreviations

EOC: Epithelial ovarian cancer
DE: Differentially expressed
DEGs: Differentially expressed genes
CCK8: Cell Counting Kit-8
qRT-PCR: RNA extraction and quantitative Real-time PCR
ELISA: Enzyme-linked immunosorbent assay
TCGA: The Cancer Genome Atlas
TME: Tumor microenvironment
DDR: DNA damage repairment
