# Supplementary figures and images for: CXCL2-mediated ATR/CHK1 signaling pathway and platinum resistance in epithelial ovarian cancer
Source: J Ovarian Res. 2021 Sep 3;14:115. doi: 10.1186/s13048-021-00864-3 (PMC8414676; doi:10.1186/s13048-021-00864-3)

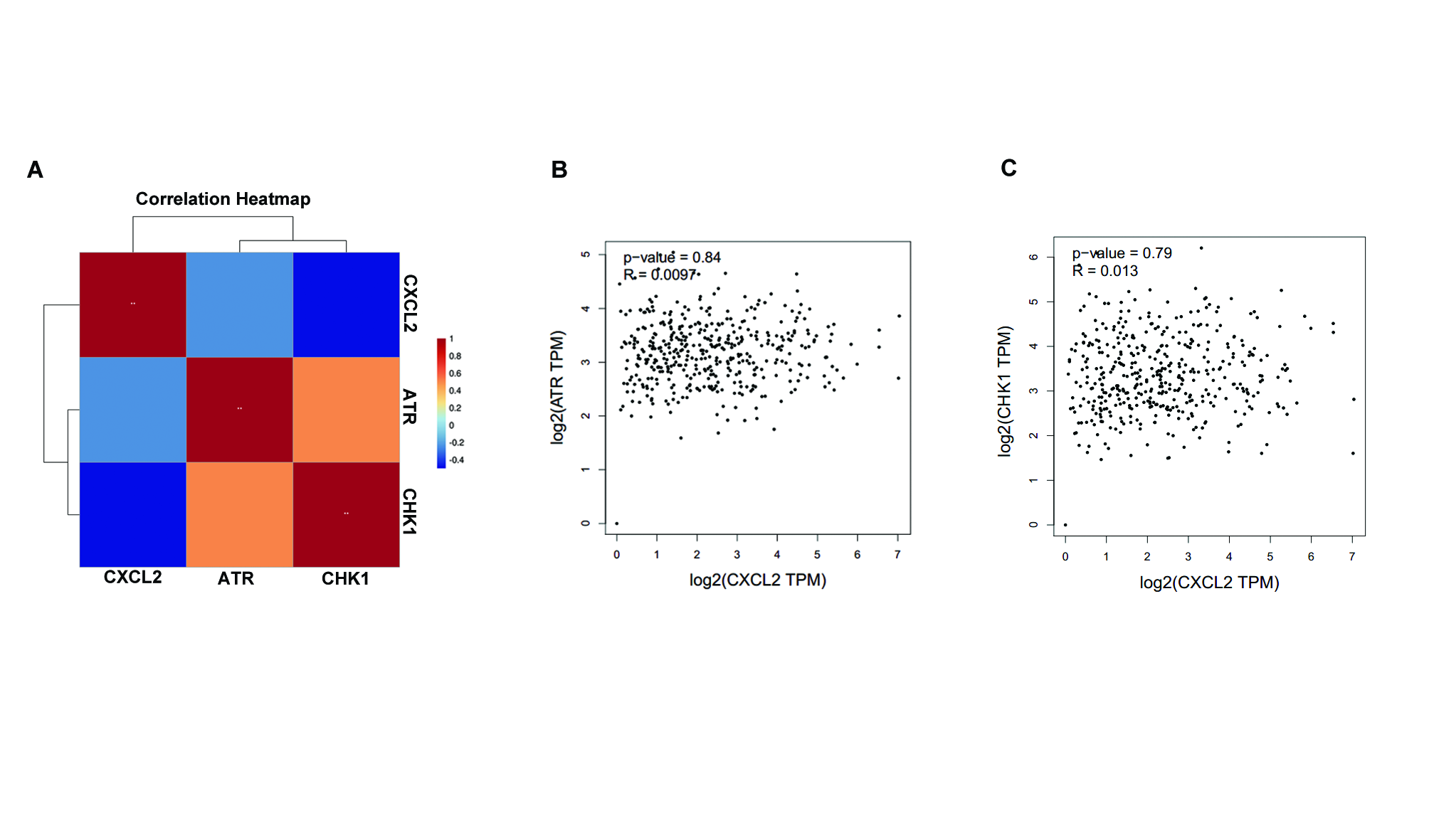

Supplement: Supplementary file 1 — Additional file 1: Supplementary Figure 1. The correlation of CXCL2 and ATR/CHK1 gene expression in EOC. (A) The correlation analyses of CXCL2 and ATR/CHK1 based on gene expression profile of GSE114206 showed no statistical significance. (B and C)The correlation analyses of CXCL2 and ATR/CHK1 based on TCGA database also showed no statistical significance. Abbreviation: TCGA: The Cancer Genome Atlas. [file 13048_2021_864_MOESM1_ESM.tif]
