# Supplementary material for: CXCL2-mediated ATR/CHK1 signaling pathway and platinum resistance in epithelial ovarian cancer
Source: J Ovarian Res. 2021 Sep 3;14:115. doi: 10.1186/s13048-021-00864-3 (PMC8414676; doi:10.1186/s13048-021-00864-3)
Supplement: Supplementary file 2 — Additional file 2: Supplementary Table 1: The sequences of gene primers used for qRT-PCR. [file 13048_2021_864_MOESM2_ESM.docx]

| Gene | Forward | Reverse |
| --- | --- | --- |
| CXCL-2 | GCTTGTCTCAACCCCGCATC | TGGATTTGCCATTTTTCAGCATCTT |
| CXCL-11 | GACGCTGTCTTTGCATAGGC | GGATTTAGGCATCGTTGTCCTTT |
| CXCL-13 | GCTTGAGGTGTAGATGTGTCC | CCCACGGGGCAAGATTTGAA |
| NANOG | CCCCAGCCTTTACTCTTCCTA | CCAGGTTGAATTGTTCCAGGTC |
| SOX2 | GCCGAGTGGAAACTTTTGTCG | GGCAGCGTGTACTTATCCTTCT |
| OCT4 | GGGAGATTGATAACTGGTGTGTT | GTGTATATCCCAGGGTGATCCTC |
| ATR | TCCCTTGAATACAGTGGCCTA | TCCTTGAAAGTACGGCAGTTC |
| CHK1 | CCAGATGCTCAGAGATTCTTCCA | TGTTCAACAAACGCTCACGATTA |
| GAPDH | GCACCGTCAAGGCTGAGAAC | GGATCTCGCTCCTGGAAGATG |

**Supplementary Table 1**

The sequences of gene primers used for qRT-PCR
