# Supplementary material for: CXCL2-mediated ATR/CHK1 signaling pathway and platinum resistance in epithelial ovarian cancer
Source: J Ovarian Res. 2021 Sep 3;14:115. doi: 10.1186/s13048-021-00864-3 (PMC8414676; doi:10.1186/s13048-021-00864-3)
Supplement: Supplementary file 3 — Additional file 3: Supplementary Table 2. [file 13048_2021_864_MOESM3_ESM.docx]

**Supplementary Table2**

| **Reagents and antibodies** | **Article number** |
| --- | --- |
| The recombinant human CXCL2 | ab268433, Abcam |
| Anti-human CXCL2 | ab89324, Abcam |
| Anti-human NANOG | ab21624, Abcam |
| Anti-human SOX2 | ab218520, Abcam |
| Anti-human OCT4 | ab200834, Abcam |
| Anti-human ATR | ab2905, Abcam |
| Anti-human CHK1 | [ab133277,](https://www.abcam.cn/chk1-antibody-2g1d5-ab69536.html) Abcam |
| Anti-human beta-Actin (β-Actin) | [ab8226,](https://www.abcam.cn/chk1-antibody-2g1d5-ab69536.html) Abcam |
| Anti-human IgG | ab188776, Abcam |
| SB225002 | 182498-32-4, MedChemExpress |
| SAR-020106 | 1184843-57-9, MedChemExpress |
